# Transperineal Focal Laser Ablation of the Prostate for Prostate Cancer: A Systematic Review of the Literature

**DOI:** 10.3390/cancers17060968

**Published:** 2025-03-13

**Authors:** Paolo Polverino, Mattia Lo Re, Luisa Moscardi, Giulio Raffaele Resta, Corso Caneschi, Francesca Conte, Beatrice Giustozzi, Anna Rivetti, Alessio Pecoraro, Vincenzo Li Marzi, Riccardo Campi, Sergio Serni, Francesco Sessa

**Affiliations:** 1 Unit of Urological Robotic Surgery and Renal Transplantation, Careggi Hospital, University of Florence, 50134 Florence, Italy; mattia.lore@unifi.it (M.L.R.); luisa.moscardi@unifi.it (L.M.); giulioraffaele.resta@unifi.it (G.R.R.); corso.caneschi@unifi.it (C.C.); francesca.conte1@unifi.it (F.C.); beatrice.giustozzi@unifi.it (B.G.); anna.rivetti@unifi.it (A.R.); alessio.pecoraro@unifi.it (A.P.); riccardo.campi@unifi.it (R.C.); sergio.serni@unifi.it (S.S.); francesco.sessa@unifi.it (F.S.); 2 Department of Experimental and Clinical Medicine, University of Florence, 50134 Florence, Italy; 3 Department of Urology, University of Siena, 53100 Siena, Italy; vlimarzi@hotmail.com

**Keywords:** prostate cancer, focal therapy, laser, focal laser ablation, TPLA

## Abstract

**Objectives**: The aim of this work was to review the available evidence on transperineal focal laser ablation (FLA) for patients with localized PCa, focusing on both functional and oncological outcomes. **Methods**: A comprehensive review of the English-language literature was performed using the MEDLINE (via PubMed) and Web of Science (WOS) databases until 30 December 2024, using a combination of free text and MeSH subject headings. The review process was carried out according to the PRISMA guidelines. **Results**: The literature search found 156 papers, and among these, 10 papers were finally accepted and included. A risk of bias assessment was conducted, revealing low-quality evidence and high heterogeneity among the available data. Low- to high-risk cancers were treated across the studies. A drop in PSA values was observed in all studies after FLA, but no definition of biochemical disease-free survival was established. The postfocal presence of cancer rate ranged from 4% to 57%, while clinically significant cancer was detected in 0 up to 31% of cases. Secondary treatments were necessary for 7 to 30%. The overall complication rate ranged from 0% to 66%, most being mild and transient. Functional outcomes appeared to be preserved both in the short- and long-term follow-ups. Quantitative analyses were not performed due to the low number and heterogeneity of the studies included. **Conclusions**: Transperineal FLA for the treatment of clinically localized prostate cancer appears to be a feasible, safe technique with an efficacy comparable to other focal therapy modalities. However, the low quality of the evidence available highlights the need for comparative, randomized long-term follow-up studies versus standard of care and other focal therapy options in order to standardize patient selection, treatment protocols, and follow-up strategies.

## Simple Summary

The standard of care for clinically localized prostate cancer (PCa) includes several options such as watchful waiting, active surveillance (AS), and whole gland treatments, which might have a non-negligible impact in terms of morbidity and quality of life. As a result, several focal therapy options have been developed to reduce the rate of side effects while ensuring good oncological outcomes and cancer control. For this scenario, transperineal laser ablation is proposed as a focal therapy option. The aim of this systematic review was to evaluate the available evidence on transperineal focal laser ablation focusing on both functional and oncological outcomes.

## 1. Introduction

The standard of care for clinically localized prostate cancer (PCa) currently includes several treatment options in light of specific patient- and tumor-related factors, such as watchful waiting, active surveillance (AS), and “whole gland” treatment techniques, particularly radiotherapy (RT) and radical prostatectomy (RP) [1]. Despite technological advancements in robotic and radiotherapy platforms, these treatments are still associated with a non-negligible impact on postoperative morbidity and quality of life, particularly concerning sexual function, incidence of cystitis, and continence [2,3,4]. For these reasons and supported by increasing scientific evidence demonstrating comparable overall survival (OS) rates among RT, RP, and AS for low- to intermediate-risk PCa, European guidelines have progressively emphasized the importance of AS. However, while AS is associated with comparable OS rates to active treatments for patients with low-risk PCa, it also carries higher rates of disease progression and metastasis, along with a considerable psychological impact on patients’ quality of life [5]. As a result, several focal therapy options have been developed and studied over the years in order to reduce the risk of progression while ensuring low rates of postoperative morbidity [6]. These technologies aim to selectively target specific areas of the prostate, particularly the index lesion [7]. The widespread adoption of multiparametric magnetic resonance imaging (mpMRI) in the diagnostic pathway for PCa, along with the subsequent development of fusion biopsy techniques, has significantly accelerated the growth of these treatment options. Among these, the strongest evidence in the literature has been reported regarding high-intensity focused ultrasound (HIFU) and cryotherapy [8,9]. Additional treatment options in this setting include light-based therapies, such as photodynamic therapy (PDT), which induces cellular damage through the production of reactive oxygen species, and photothermal therapy (PTT), where hyperthermia generated by the light beam directly causes tumor cell destruction. These treatments can be enhanced by the use of nanoparticles, particularly gold nanoparticles (AuNPs), which act as “amplifiers” of the light beam at the tumor tissue level [10].

The advantages of these techniques are undoubtedly linked to their minimally invasive nature, which significantly reduces hospitalization time compared to traditional techniques, lowers the rate of sexual dysfunction-related complications, and decreases perioperative adverse events such as bleeding. However, long-term studies on both functional and oncological outcomes are lacking. For this reason, European guidelines recommend their use only within well-designed prospective trial settings [1].

In this scenario, although transperineal laser ablation has been used for many years to treat benign prostatic hyperplasia [11,12], it has recently been proposed as a new focal therapy option for patients with localized PCa.

This systematic review aimed to evaluate the available evidence on transperineal focal laser ablation (FLA) for patients with localized PCa, focusing on both functional and oncological outcomes.

## 2. Materials and Methods

This systematic review was carried out according to the Preferred Reporting Items for Systematic Reviews and Meta-analyses (PRISMA) guidelines [13], and the review protocol was registered in PROSPERO (CRD42024616173).

A comprehensive review of the English-language literature was conducted using the MEDLINE (via PubMed) and Web of Science (WOS) databases up to 30 December 2024. The search strategy combined free text and MeSH terms to identify prospective or retrospective studies assessing the outcomes of transperineal FLA in patients with localized prostate cancer (Appendix A). Additional records were identified through a reference screening of the retrieved studies and manual searches in Google Scholar. Data from the included studies were extracted using pre-defined data extraction forms and are summarized in four tables.

### 2.1. Selection Criteria

The PICOS (Patient, Intervention, Comparison, Outcomes, Study design) model was used:

P: Patients with Localized PCa,
I: Focal therapy using transperineal laser ablation of the prostate,
C: Single-arm or comparative studies,
O: Oncological outcomes,
S: Prospective or retrospective studies.

### 2.2. Study Screening and Selection

Studies were accepted based on PICOS eligibility criteria. Preclinical and animal studies were excluded. Ablations performed with a transrectal approach were excluded. Reviews, letters to the editor, case reports, and meeting abstracts were also excluded. Only English papers were accepted. Retrospective, prospective, and prospective randomized studies were accepted.

All retrieved studies were screened by two independent authors (L.M., G.R.R.). A third author (P.P.) solved discrepancies through a discussion. The full text of the screened papers was selected if found pertinent to the aim of this review.

From each study, the following data were extracted: (1) study characteristics; (2) laser system; (3) patient characteristics such as median age, retrieval of preoperative biopsy, imaging, PSA, Gleason score, and risk classification; (4) type of ablation; (5) follow-up; (6) oncological outcomes (such as postprocedural PSA level, percentage of positive biopsies, failure rates, and overall survival); (7) adverse events; and (8) functional outcomes (patient-reported outcomes and questionnaire scores).

### 2.3. Risk of Bias Assessment

Risk of bias was independently assessed by two authors (L.M., G.R.R.) using the ROB 2.0 tool, which evaluates bias and applicability across five key domains. In the event of disagreement, a third author (P.P.) was involved to resolve the issue.

## 3. Results

### 3.1. Characteristics of the Included Studies

**Figure 1:**
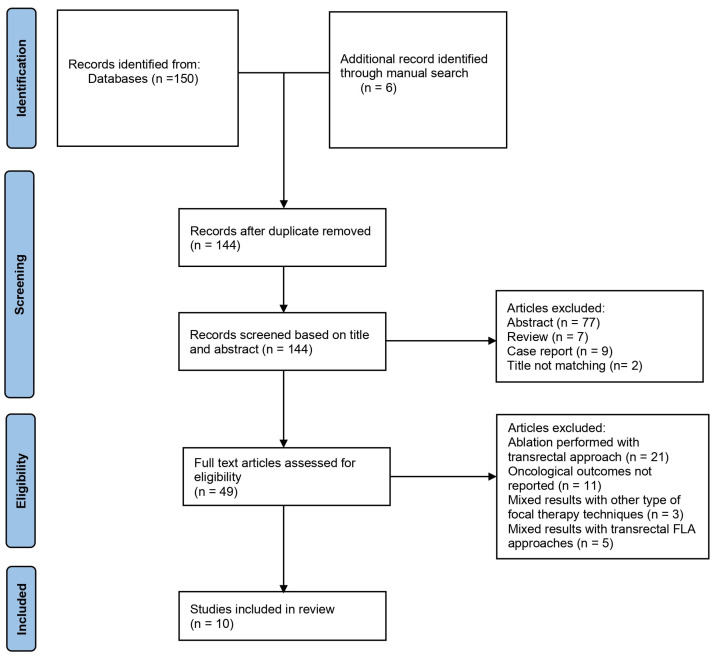
PRISMA flowchart.

The literature search found 156 papers. After removing 12 duplicates, 144 papers were screened against the title, abstract, and type of article. Among these, 95 papers were further excluded for not aligning with the scope of this review. The remaining 49 full-text papers were screened for eligibility, and 10 papers were finally accepted and included [14,15,16,17,18,19,20,21,22,23]. A flowchart outlining the overall review process in accordance with PRISMA guidelines is presented in Figure 1.

The key characteristics of the studies contained in the review are reported in Table 1. Overall, ten studies from different countries (three from Italy and USA, two from Canada, one from Germany and France) conducted between 2009 and 2024 were included in our review [14,15,16,17,18,19,20,21,22,23]. Of these, nine studies were prospective, while one was retrospective [20]. All the studies were single-arm, non-comparative, monocentric studies. The number of patients across the included studies ranged between 7 and 55.

### 3.2. Risk of Bias

**Figure 2:**
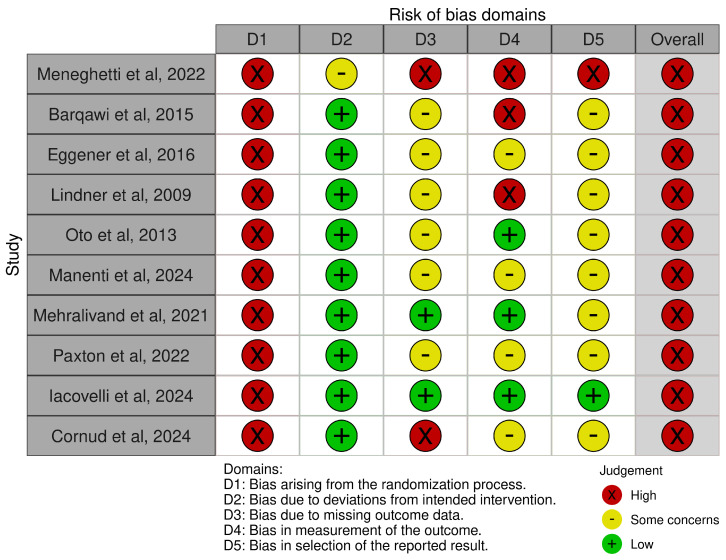
Risk of bias assessment [14,15,16,17,18,19,20,21,22,23].

The proportion of studies with a low risk of bias in randomization, deviation from intended intervention, missing data, outcome measurement, and selection of reported results domains was 0%, 90%, 20%, 30%, and 10%, respectively (Figure 2). All the studies included had an overall high risk of bias, prompting a judicious interpretation of the review findings. In particular, only a minority of studies reported the outcome measures with a low risk of bias, and there were at least some concerns in the selection of the reported results in almost all studies.

### 3.3. Preoperative Characteristics of Patients

Table 1 provides a comprehensive overview of the preoperative characteristics of the patients included in the studies analyzed in this systematic review.

**Table 1:** Preoperative characteristics.

Study	Meneghetti et al., 2022 [14]	Barqawi et al., 2014 [15]	Eggener et al., 2016 [16]	Lindner et al., 2009 [17]	Oto et al., 2013 [18]	Manenti et al., 2024 [19]	Mehralivand et al., 2021 [20]		Paxton et al., 2022 [21]	Iacovelli et al., 2024 [22]	Cornud et al., 2024 [23]
**Type and design**	Monocentric prospective non-comparative	Monocentric prospective non-comparative	Monocentric prospective non-comparative	Monocentric prospective non-comparative	Monocentric prospective non-comparative	Monocentric prospective non-comparative	Monocentric prospective Non-comparative		Monocentric retrospective non-comparative	Monocentric prospective non-comparative	Monocentric prospective non-comparative
**Country**	Italy	USA	USA	Canada	USA	Italy	Germany		Canada	Italy	France
**Patients, no**	10	7	27	12	9	25	15		54	24	55
**Age, yr**	Range 60–78	Range 56–69	Median: 62	Median: 56.5 (range 51–62)	Median: 61 (range 52–77)	Range 46–86	Median: 66 (range 47–75)		Mean: 61 (range 43.1–77)	Median: 67 (SD ± 12)	median 70 (IQR 62–74)
**Pre- operative Imaging**	MRI	MRI	MRI	MRI	MRI	MRI	MRI		MRI	MRI	mpMRI, microUS
**Pre- operative Biopsy**	Transperineal (systematic or cognitive)	Transperineal (3DMB systematic)	NR	Transrectal (systematic)	Transrectal (systematic)	NR	Transrectal (target)		Transrectal (target)	Transperineal (systematic and target)	MRI-MicroUS-guided biopsies (systematic + targeted)
**Location**	NR	7 (36.8%):PZ; 1 (5.3%): junction of PZ and central gland; 11 (57.9%): TZ	NR	NR	NR	NR	16 lesions: 3 R mid-anterior TZ, 1 L apical anterior PZ, 2 R base PZ, 1 R mid TZ, 1 L apical anterior TZ, 1 R apical	anterior PZ, 1 L mid-base anterior TZ, 1 M mid-base TZ, 2 L apical PZ, 1 M apical anterior PZ, 1 R apical anterior TZ, 1 R apical-mid anterior TZ	51 (92.7%): PZ, 4 (7.3%): TZ	9 (37.5%): posterior PZ; 6 (25%): anterior zone o TZ; 9 222 (37.5%): apical zone	58 lesions: 23 (39.5%) PZpm, 7 (12%) PZpl, 5 (8.5%) PZa; 1 (2%) TZpm, 22 (38%) TZpl. 11 (19%) apex, 42 (72.5%) mid, 5 (8.5%) base
**PSA level, ng/mL**	Mean: 7.9 (SD ± 4.1; range 5.1–17.8)	Mean: 5.05 (SD ± 0.89)	Mean: 4.4 (range 0.88–8.99)	Mean 5.7 (SD ± 1.1)	Mean: 5.5	Range: 5.5–6.9	Median: 6.19 (range 2.16–14.5)		Mean: 5.44 (range 0.11–18.31)	Median: 5.7 (SD ± 3.9)	Median 7 (IQR 5.6–9)
**Gleason Score**	4 (40%): 3 + 3; 2 (20%): 3 + 4; 3 (30%): 4 + 3; 1 (10%): 4 + 4	7 (100%): 3 + 3	23 (85%): 3 + 3, 3 (11%): 3 + 4, 1 (4%): 4 + 3	12 (100%): 3 + 3	NR	NR	6 (40%): 3 + 3; 9 (60%): 3 + 4		33 (60%): 3 + 3, 21 (38%): 3 + 4; 1 (2%): 4 + 3	14 (58.3%): 3 + 3; 10 (41.7%): 3 + 4	10 (17%): 3 + 3; 43 (74%) 3 + 4; 3 (5.5%) 4 + 3; 2 (3.5%) 4 + 4
**Risk Classification (D’Amico)**	4 (40%): Low; 5 (50%): Intermediate; 1 (10%) High	7 (100%): Low	NR	12 (100%): Low	NR	NR	6 (40%): Low, 9 (60%): Intermediate		NR	NR	NR

NR: not reported; MRI: Magnetic Resonance Imaging; 3DMB: 3D mapping biopsy; TZ: transitional zone; PZ: peripheral zone.

The age of patients included in the studies ranged from a minimum of 43 to a maximum of 86 years. In all studies, patients underwent preliminary evaluation with multiparametric magnetic resonance imaging (mpMRI). In addition to this assessment, Cornud et al. also integrated prostate ultrasound using the ExactVu micro-ultrasound system (Exact Imaging, Markham, Ontario, Canada) [23]. The mean PSA values reported across the studies ranged from 4.4 to 7.9 ng/mL. Regarding the preoperative histological diagnosis of PCa, the biopsies in the various studies differed in both approach (transrectal vs. transperineal) and technique (targeted vs. systematic biopsy). Specifically, three authors used a transperineal approach [14,15,22], while four employed a transrectal approach [17,18,20,21]; moreover, in three studies, the chosen biopsy approach was not specified [16,18,23]. Additionally, only Cornud et al. and Iacovelli et al., in their studies, performed both targeted and systematic biopsies [22,23]. In five studies, the lesion locations were reported, and in all cases, both peripheral zone and transitional zone lesions were included and treated [15,20,21,22,23]. The Gleason score of the treated lesions varied across studies. Specifically, two authors treated only ISUP 1 tumors [15,17], while another two included both ISUP 1 and ISUP 2 [20,22]. Two studies treated tumors ranging from ISUP 1 to ISUP 3 [16,21], and another two extended to ISUP 4 [14,23]. Lastly, two authors did not report the Gleason scores of the treated lesions [18,19].

### 3.4. Technical Aspects

A detailed overview of operative features is shown in Table 2.

The procedure was performed under local anesthesia in three cases, with conscious sedation also administered in two studies [18,19,20,21,22]. Eggener et al. and Paxton et al. reported using sedation alone [16,21]. Additionally, three authors indicated that the procedure was carried out under general anesthesia [15,17,23], while Mehralivand et al. and Meneghetti et al. did not specify the type of anesthesia used [14,20]. Only four authors provided information regarding hospitalization times, with most procedures performed as day surgeries, allowing patients to be discharged on the same day as the procedure [15,17,19,23]. In all studies, the procedures were performed as focal ablations, with no reports of hemiablations or whole-gland ablations. The treatments were performed using various laser systems. In all cases, diode lasers with wavelengths in the infrared range (between 980 and 1064 nm) were used, offering the ability to penetrate tissues and generate heat in a controlled manner. Specifically, the Echolaser system (Elesta, Calenzano, Italy) was used in four studies, the Visualase system (Houston, TX, USA) in five studies, and the Indigo Optima system (Ethicon Endo-Surgery, Inc., Cincinnati, OH, USA) in one study. A significant variability among the studies can also be observed in terms of treatment protocols, particularly regarding the fibers used. Oto et al. employed a standard protocol with single-fiber ablation [18], whereas other authors determined the number of fibers based on the volume and number of areas to be treated, using up to five fibers per patient, as reported in the study by Cornud et al. [23].

### 3.5. Oncological Outcomes

The studies analyzed showed variable rates of oncological effectiveness after focal laser ablation for prostate cancer (Table 3).

The postfocal presence of cancer rate ranged from 4% to 57% based on follow-up biopsies [14,15,16,17,18,19,20,21,22,23], while clinically significant cancer was reported in 8/10 studies, detected in 0 up to 31% of cases [16,17,18,19,20,21,22,23]. Biochemical disease-free survival (bDFS) rates were not consistently reported. Treatment failure was defined in 4/10 studies: Lindner, Manenti, and Iacovelli [17,19,22] agreed on defining it as the presence of residual lesions within the treatment field, while Meneghetti [14] described it as positive MRI or biopsy at follow-up.

PSA kinetics generally indicated significant reductions, suggesting effective tumor targeting, though variations existed depending on the treatment protocol and patient selection; Bargawi and Mebralivand [15,20] reported a statistically significant reduction of PSA at follow-up.

Secondary treatments, including radical prostatectomy and focal re-ablations, were necessary in a subset of patients, primarily due to residual disease. Secondary treatment ranged from 7 to 30% [14,16,17,20,21,22,23]; Meneghetti [14] recorded a higher rate of secondary treatment, with three patients (30%) who underwent re-FLA; in single cases of recurrence, Eggener and Lindner [16,17] performed radical prostatectomy, while Mebralivand [20] performed two re—FLA, one radical Prostatectomy and one radical prostatectomy followed by androgen deprivation therapy (ADT) and enzalutamide. Only Iacovelli [22] in two (8.3%) cases performed radiotherapy as a secondary treatment.

### 3.6. Functional Outcomes

Table 4 provides detailed information on the functional outcomes and safety data reported in the studies included in this review.

### 3.7. Complications

Complication rates varied, with overall rates ranging from 0% to 66.6%, as shown in Table 4 [14,15,18,19,20,22,23]. Most of the authors reported Clavien Dindo I and II complications while most common complications included mild hematuria, transient perineal pain or discomfort, and urinary retention. Mehralivand et al. [20] reported three cases of urinary tract infections, such as cystitis, prostatitis, or epididymitis. Cornud et al. [23] reported the only severe complications, such as rectoprostatic fistula, treated with surgery. Almost all the authors reported day hospital procedures; only Lindner [17] reported three (25%) cases of one day of hospitalization due to mild complications.

### 3.8. Urinary and Sexual Function

Urinary continence outcomes were reported in 4/10 studies [14,16,20,23] with most patients maintaining complete continence (0% pads required) post-treatment; Iacovelli [22] reported transient urgency in seven (29.2%) patients in the first 72 h, which completely regressed at follow-up. Lower urinary tract symptom (LUTS) was evaluated with the International Prostate Symptoms Score (IPSS) in 8/10 studies [14,16,17,18,19,20,22,23], showing no significant changes across most studies.

Erectile function was alternatively evaluated with the International Index of Erectile Function (IIEF-5) [14,17,19,22,23] or Sexual Health Inventory for Men (SHIM) [15,16,18,20] scores. In almost the totality of the studies, no significant changes where reported [14,17,18,19,20]. Eggener et al. reported a decrease in SHIM after 1 and 3 months (*p* = 0.03 and *p* = 0.05, respectively), with a return to values comparable to preoperative levels at 12 months (*p* = 0.38); Cornud et al. was the only author reporting a statistically significant persistent improvement in the IIEF-5 value at 6 and 12 months (19 vs. 22, *p* < 0.001, and 19 vs. 21, *p* < 0.001, respectively).

## 4. Discussion

The studies analyzed in this systematic review suggest that transperineal focal ablation for prostate cancer is a safe and effective therapeutic approach. However, the available evidence remains limited, with substantial heterogeneity in indications, treatment modalities, and follow-up protocols. To the best of our knowledge, this is the first systematic review evaluating data on FLA for localized PCa performed with an exclusive transperineal approach.

In this context, our review provides valuable insights for clinicians, patients, and stakeholders. First, the reported complication rates varied widely, ranging from 0% to 66%, though most complications were mild (Clavien–Dindo grade I or II) and transient. Only one major complication—a rectoprostatic fistula requiring surgical treatment—was reported in the study by Cornud et al. [23]. Notably, the overall incidence of complications was comparable to that observed with other focal therapy modalities, such as HIFU [24].

Second, our findings suggest favorable postoperative functional outcomes following transperineal FLA. A 2016 Delphi Consensus on focal therapy for prostate cancer defined functional success as the preservation of voiding patterns, erectile function, and quality of life at 12 months post-treatment, assessed using the IIEF-5 and IPSS scores [25]. In our review, these functions appear to be preserved both in the short and long terms across all reported studies, except for a few cases of temporary urge incontinence in the immediate post-treatment period. However, functional outcomes were reported inconsistently, as different studies used varying assessment tools (IPSS and AUA-SS for LUTS, IIEF-5 and SHIM for erectile function), complicating direct comparisons. Comparing sexual outcomes with other focal therapy modalities, the studies included in this review suggest a higher rate of postoperative erectile function preservation. Specifically, when compared to data on HIFU and, particularly, cryotherapy from a recent systematic review, transperineal focal laser ablation appears to show more favorable functional outcomes. Similarly, in terms of urinary symptoms, our systematic review suggests a lower incidence of postoperative incontinence and urinary retention [24].

Furthermore, significant variability was observed in patient selection and tumor characteristics, with two studies including patients with ISUP 4 tumors. This finding is consistent with results from other systematic reviews on focal therapy modalities [26].

Finally, the central point emerging from this systematic review concerns the available data on the effectiveness of transperineal focal laser ablation of the prostate, particularly in terms of biochemical disease-free survival (bDFS), therapeutic failure, and, consequently, cancer control. Cancer control is closely linked to the concept of biochemical recurrence (BCR), a well-established criterion widely used in clinical practice to evaluate cancer control following treatments for clinically localized prostate cancer, such as prostatectomy and radiotherapy. Specifically, after prostatectomy, the reference value for BCR is 0.2 ng/mL, while the Phoenix criteria are applied post-radiotherapy [27]. In the context of focal therapy, while some reference values have been proposed for follow-up after HIFU treatment, there is a significant lack of uniformity, or even an absence of clear and standardized definitions and cutoffs for evaluating PSA changes following focal therapy [28,29]. This is evident in the studies included in our review, where no criteria for biochemical disease-free survival are defined, and consequently, no corresponding rates are reported. Similarly, the studies analyzed do not present a consistent approach to defining treatment failure, with six out of ten studies not providing any definitions at all. The remaining studies primarily use recurrence at the treatment site as the criterion to define therapeutic failure, with failure rates ranging from 12.5% to 30%. This finding is consistent with data reported in the literature for other focal therapy modalities, as highlighted in a recent systematic review, particularly for HIFU and cryotherapy [26]. However, it is important to note that the studies included in our review generally have smaller sample sizes and shorter follow-up periods, which may limit the strength of these comparisons.

The heterogeneity in patient selection and outcome reporting underscores the need for standardized criteria to better define the role of transperineal focal ablation in clinical practice. Therefore, our review highlights the urgent need to standardize follow-up protocols and establish well-defined postoperative outcomes to assess the current role of transperineal FLA, including clear definitions of “treatment failure”, “recurrence”, and optimal functional results.

Our review is not devoid of limitations. In particular, quantitative analysis was not performed due to the relatively low number of patients, the heterogeneity of the included studies, and the lack of randomized clinical trials and/or comparative studies. Moreover, the absence of a standardized and widely accepted definition of endpoints represents a significant limitation of this review, hindering direct comparisons between studies. At the study level, most included studies were constrained by small sample sizes, heterogeneous selection criteria, and short follow-up periods. Additionally, our review was limited to studies published in English. Therefore, our findings should be interpreted with caution, particularly concerning the mid- and long-term safety and oncological outcomes of transperineal FLA.

## 5. Conclusions

Transperineal FLA for clinically localized prostate cancer appears to be a feasible and safe technique, offering oncological and functional outcomes comparable to other focal therapy modalities. However, the available evidence remains limited, underscoring the need for well-designed comparative studies against the standard of care. Additionally, standardizing patient selection criteria, treatment protocols, and follow-up strategies is essential to optimizing clinical outcomes and establishing its role in routine practice.

## Figures and Tables

**Table 2:** Operative features.

Study	Meneghetti et al., 2022 [14]	Barqawi et al., 2014 [15]	Eggener et al., 2016 [16]	Lindner et al., 2009 [17]	Oto et al., 2013 [18]	Manenti et al., 2024 [19]	Mehralivand et al., 2021 [20]	Paxton et al., 2022 [21]	Iacovelli et al., 2024 [22]	Cornud et al., 2024 [23]
**Laser**	Echolaser	Visualase	Visualase	Indigo Optima laser	Visualase	Echolaser	Visualase	Visualase	Echolaser	Echolaser
**Type of ablation**	Focal ablation	Focal ablation (real-time MRI-guided)	Focal ablation	Focal ablation	Focal ablation (MRI-guided)	Focal ablation (US/MRI-guided)	Focal ablation (real-time MRI-guided)	Focal ablation (real-time MRI-guided)	Focal ablation	Focal ablation
**Number of fibers**	NR	1 to 3 (depending on the number of cancer foci)	1 to 2 (occasionally, a second laser fiber was required to complete the ablation)	1 to 2 (depending on the volume of the lesion)	9 (100%): 1	1 to 3	NR	NR	18 (75.0%): 1; 6 (25.0%): 2	21 (36%): 1; 24 (41%): 2; 6 (10.5%): 3; 6 (10.5%): 4; 1 (2%): 5
**Length of stay (days)**	NR	7 (100%): 0 (Day Surgery)	NR	9 (75%): 0; 3 (25%): 1	NR	25 (100%): 0 (Day Surgery)	NR	NR	NR	55 (100%): 0
**Anesthesia**	NR	General anesthesia	Conscious sedation	General anesthesia	Conscious sedation and local anesthesia	Local anesthesia	NR	Deep sedation (intravenous Propofol)	Conscious sedation and local anesthesia (anesthesia of perineal skin and peri-prostatic nerve-block)	General anesthesia

NR: not reported; MRI: Magnetic Resonance Imaging; US: Ultrasound.

**Table 3:** Oncological outcomes.

Study	Meneghetti et al., 2022 [14]	Barqawi et al., 2014 [15]	Eggener et al., 2016 [16]	Lindner et al., 2009 [17]	Oto et al., 2013 [18]	Manenti et al., 2024 [19]	Mehralivand et al., 2021 [20]	Paxton et al., 2022 [21]	Iacovelli et al., 2024 [22]	Cornud et al., 2024 [23]
**Follow-up time (months)**	12	12	12	6	6	36	36	Median: 49 (range 21–101)	12	12
**Postfocal imaging**	CEUS (1 h); MRI (3 and 12 months)	MRI	MRI	CEUS (7 days); MRI (7 days)	MRI	MRI	MRI	MRI	MRI	MRI
**Postfocal histology (reason)**	8 (80%): systematic biopsy (by protocol); 2 (20%): refused	5 (71%): 12 core TRUS biopsy; 1 (14%): 3DMB (by protocol); 1 (14%): lost to follow-up	27 (100%): MRI-guided biopsy at 3 months (2 cores) and systematic biopsy at 12 months (by protocol)	12 (100%): systematic prostate biopsies at 3 and 6 months (10 cores) (by protocol)	9 (100%): MRI-guided biopsy (by protocol)	25 (100%): systematic and target MRI/US fusion biopsy (by protocol)	15 (100%): systematic and target MRI/US fusion biopsy at 12 and 24 months (by protocol); 12 (80%): systematic and target MRI/US fusion biopsy at 36 months (optional by protocol)	51 (93%) targeted at early f-up (median 4 months), 21 (18.5%): systematic and target biopsy at late f-up (median 49 months) (by protocol)	24 (100%): fusion biopsy at 12 months (by protocol)	33 (60%) fusion re-biopsy (by protocol) at 12 months
**Postfocal presence of cancer rate** **(%)**	2 (20%)	4 (57%)	1 (4%) at 3 months, 10 (37%) at 12 months	6 (50%)	2 (22%)	5 (20%)	7 (46.6%): residual cancer; 2 (13.3%): cancer outside the treatment area	5 (9.2%) at early f-up, 8 (14.8%) at late f-up	7 (29.2%)	17/35 (49%)
**Clinically significant cancer**	NR	NR	0 (0%) at 3 months; 2 (7%) at 12 months	0 (0%)	0 (0%)	5 (20%)	2 (13%)	6 (11%)	1 (4.2%)	11/35 (31%)
**bDFS, %**	NR	NR	NR	NR	NR	NR	NR	NR	NR	NR
**Criteria for bDFS**	NR	NR	NR	NR	NR	NR	NR	NR	NR	NR
**Treatment failure rate**	3 (30%)	NR	NR	4 (30%)	NR	5 (20%)	NR	NR	3 (12.5%)	NR
**Treatment failure definition**	Positive MRI or biopsy	NR	NR	Any cancer in ablated zone	NR	Presence of residual lesions within the treatment field	NR	NR	Recurrence in the target treated lesion at 12 months	NR
**PSA kinetics (at last follow-up unless otherwise stated)**	3.7 (SD ± 1.1)	3.94 (SD ± 1.68) (*p* value = 0.149)	3.4 (SD ± 2.2) (not significant)	NR	no changes in PSA level observed after treatment	decrease higher than 87% in all the patients except for the five who experimented with treatment failure	PSA level was significantly lower at follow-up than at baseline (*p* < 0.001)\	Mean 6.4 (range 1.0–16.4)	Median 3.7 (SD ± 2.7) (*p* value: 0.9)	Single fiber median 3.4 (1.7–4.4); multifiber median 2.8 (2.3–4.5)
**Secondary treatment**	3 (30%): FLA	NR	1 (7%): RP	1 (8.3%): RP	NR	NR	2 (13%) FLA; 1 (7%) RP; 1 (7%) RP + neoadjuvant Enzalutamide + androgen deprivation therapy	5 (9.2%): whole gland treatment (not specified)	4 (16.7%): RP; 2 (8.3%) EBRT; 1 (4.2%): TPLA	6 (11%): FLA; 3 (5.5%): RP
**Mortality**	0 (0%)	0 (0%)	0 (0%)	0 (0%)	0 (0%)	0 (0%)	0 (0%)	0 (0%)	0 (0%)	0 (0%)

NR: not reported; TRUS: Transrectal Ultrasound; MRI: Magnetic Resonance Imaging; CEUS: Contrast Enhancement Ultra Sonography; FLA: Focal laser ablation; RP: radical prostatectomy; TPLA: transperineal laser ablation; EBRT: External beam radiation therapy.

**Table 4:** Functional Outcomes.

Study	Meneghetti et al., 2022 [14]	Barqawi et al., 2014 [15]	Eggener et al., 2016 [16]	Lindner et al., 2009 [17]	Oto et al., 2013 [18]	Manenti et al., 2024 [19]	Mehralivand et al., 2021 [20]	Paxton et al., 2022 [21]	Iacovelli et al., 2024 [22]	Cornud et al., 2024 [23]
**Overall complication rate**	4 (40%)	1 (14%)	NR	NR	2 (22%)	0 (0%)	10 (66.6%)	NR	0 (0%)	11 (20%)
**Complication (description)**	4 (40%): pain	1 (14%): urinary retention	4 (15%): hematuria, 3 (11%) perineal ecchymosis, 2 (8%): acute urinary retention	3 (25%) perineal discomfort; 2 mild hematuria (that did not require any intervention); 2 hematospermia; 1 fatigue	1 (11.1%): abrasion on the perineum; 1 (11.1%): transient paresthesia	-	2 (13.3%) hematuria; 1 (6.6%) urgency problem; 1 (6.6%) UTI; 1 (6.6%) hematuria and bladder spasm; 1 (6.6%) pressure ulcer and gross hematuria; 1 (6.6%) UTI with need for antibiotic therapy for 4 weeks; 1 (6.6%) bilateral epididymitis and gross hematuria; 1 (6.6%) LUTS; 1 (6.6%) acute prostatitis and need for catheterization	NR	-	1 (2%) rectoprostatic fistula; 2 (3.5%) retrograde ejaculation; urinary retention; transient stress urinary incontinence; urinary tract infections
**Urinary continence**	0 (0%) (0 pad)	NR	1 (4%): urge incontinence at 1 month (resolved by 3 months)	NR	NR	NR	0 (0%) (0 pad)	NR	7 (29.2%): transient urgency (<72 h) Median ICIQ-SF: 3 months: 0 (SD ± 4) (*p* value 0.04); 6 months; 0 (SD ± 3) (*p* value 0.8); 12 months: 0 (SD ± 4) (*p* value 0.9)	NR
**LUTS**	IPSS: no changes	AUA-SS: data missing	IPSS: no changes	IPSS: no changes	IPSS: no significant changes	IPSS: no changes	IPSS: no changes	NR	Median IPSS: 3 months: 7 (SD ± 5) (*p* value: 0.009); 6 months: 7 (SD ± 6) (*p* value 0.7); 12 months: 6 (SD ± 6) (*p* value 0.3)	IPSS: no changes at 6 and 12 months
**Erectile function**	IIEF-5: no changes	SHIM: data missing	SHIM: lower at 1 month (*p* = 0.03), marginally lower at 3 months (*p* = 0.05), not significant at 12 months (*p* = 0.38)	IIEF-5: no changes	SHIM: no significant changes	IIEF-5: no changes	SHIM: no changes	NR	Median IIEF 5: 3 months: 20 (SD ± 7) (*p* value 0.2); 6 months: 21 (SD ± 6) (*p* value 0.5); 12 months: 20 (SD ± 6) (*p* value 0.8)	IIEF-5: at 6 months: 33 patients, median 19 vs. 22 *p* < 0.001; at 12 months: 29 patients, median 19 vs. 21 *p* < 0.001
**Quality of life**	NR	NR	NR	NR	NR	NR	QoL: no changes	NR	Median IPSS QoL: 3 months: 1 (SD ± 2) (*p* value 0.02); 6 months: 1 (SD ± 2) (*p* value 0.8); 12 months: 1 (SD ± 1) (*p* value 0.1)	IPSS QoL: no changes at 6 and 12 months

NR: not reported; UTI: urinary tract infection; LUTS: lower urinary tract symptoms; IPSS: International Prostatic Symptoms Score; AUA-SS: American Urological Association Symptom Score; IIEF-5: International Index of Erectile Function—5 questions; SHIM: Sexual Health Inventory for Men.

## Data Availability

No new data were created or analyzed in this study. Data sharing is not applicable to this article.

## Abbreviations

The following abbreviations are used in this manuscript:

PCa	Prostate Cancer
AS	Active Surveillance
RT	Radiotherapy
RP	Radical Prostatectomy
OS	Overall Survival
mpMRI	Multiparametric Magnetic Resonance Imaging
HIFU	High-Intensity Focused Ultrasound
FLA	Focal Laser Ablation
PRISMA	Preferred Reporting Items for Systematic Reviews and Meta-analyses
ISUP	International Society of Urological Pathology
bDFS	Biochemical Disease-Free Survival
ADT	Androgen Deprivation Therapy
LUTS	Lower Urinary Tract Symptoms
IPSS	International Prostatic Symptoms Score
IIEF-5	Index of Erectile Function 5-items
SHIM	Sexual Health Inventory for Men
BCR	Biochemical Recurrence

## Appendix A

### Appendix A.1. Search Strategy

#### Appendix A.1.1. Medline

(TRANSPERINEAL PROSTATIC LASER ABLATION OR TPLA OR “transperineal laser ablat*”[tiab] OR “transperineal laser”[TIAB:~3] OR ((transperin*[tiab] OR trans-perin*) AND laser*[tiab]) OR “Transperineal Percutaneous LaserAblation” OR TPLA OR “focal laser ablat*”[tiab] OR fla OR ”focal laser” [TIAB:~3]) AND (Prostate cancer OR (prostat*[tiab] AND (cancer*[tiab] OR tumor*[tiab] OR tumour*[tiab] OR carcinoma*[tiab] OR adenocarcinoma*[tiab] OR sarcoma*[tiab])))

#### Appendix A.1.2. Web of Science

TS = (“TRANSPERINEAL PROSTATIC LASER ABLATION” OR TPLA OR “transperineal laser ablat*” OR “transperineal laser” NEAR/3 TIAB OR ((transperin* OR trans-perin*) AND laser*) OR “Transperineal Percutaneous Laser Ablation” OR “focal laser ablat*” OR FLA OR “focal laser” NEAR/3 TIAB) AND TS = (“Prostate cancer” OR (prostat* AND (cancer* OR tumor* OR tumour* OR carcinoma* OR adenocarcinoma* OR sarcoma*)))
